# Evaluation of the Effect of Fiber Type, Length, and Content on Asphalt Properties and Asphalt Mixture Performance

**DOI:** 10.3390/ma13071556

**Published:** 2020-03-27

**Authors:** Fucheng Guo, Rui Li, Shuhua Lu, Yanqiu Bi, Haiqi He

**Affiliations:** 1 Key Laboratory for Special Area Highway Engineering of Ministry of Education, Chang’an University, Xi’an 710064, Shaanxi, China; fcguo@chd.edu.cn (F.G.); lianghw2@126.com (S.L.); hehaiqi@chd.edu.cn (H.H.); 2 Shanxi Provincial Research Institute of Communications, Taiyuan 030006, Shanxi, China

**Keywords:** fiber type, fiber length, fiber content, asphalt binder, asphalt mixture, engineering properties

## Abstract

Fiber-reinforced asphalt mixture has been widely used in pavement engineering to not only prevent asphalt binder leakage but also improve engineering properties of asphalt mixture. However, the research on three key parameters, namely fiber type, fiber length, and fiber content, which significantly affect the performance of fiber-reinforced asphalt mixture, have seldom been conducted systematically. To determine these three key parameters in the support of the application of fibers in mixture scientifically, three commonly used fibers were selected, basalt fiber, polyester fiber, and lignin fiber, and the testing on fibers, fiber-reinforced asphalt binders, and fiber-reinforced asphalt mixtures was conducted afterwards. The results showed: the favorable fiber type was basalt fiber; the favorable basalt fiber length was 6mm; the engineering properties including high temperature stability, low temperature crack resistance, and water susceptibility were clearly improved by the added basalt fiber, and the optimum basalt fiber content was 0.4 wt.%. The obtained results may be valuable from a practical point of view to engineers and practitioners.

## 1. Introduction

Asphalt pavement, as the major type of pavement, has been widely constructed in highway engineering for its good performance in improving flexibility, enhancing skid resistance, weakening stress concentration, reducing noise and dust [1]. However, asphalt pavement is prone to some premature failures, such as rutting (especially in summer) and cracking (especially in winter) [2]. Therefore, the performance of asphalt mixture needs to be improved and reinforced, where some modifiers include the polymer-modified materials and the fiber-modified materials are commonly used in asphalt mixtures [3,4,5].

Considering that the poor stability properties of polymer-modified material requires to be further enhanced, and the polymers are more difficult to decompose and result in environmental pollution when they are wasted, the fiber modified materials, especially inorganic fibers, are the more favorable modifiers and are commonly applied [6]. The addition of fibers does not only improve the properties of asphalt binder by adsorbing asphalt binder to avoid the bleeding of asphalt pavement in summer, but also improves the engineering properties of asphalt mixtures including viscoelasticity, dynamic modulus, moisture susceptibility, creep compliance, rutting resistance, and freeze–thaw resistance [7,8]. Thus, the service life of asphalt pavement can be prolonged for these benefits.

There are several fibers have been used in asphalt pavement, such as basalt fiber, lignin fiber, polyester fiber, asbestos fiber, carbon fiber, diatomite fiber, and more [7], whereas basalt fiber, lignin fiber, polyester fiber, and asbestos fiber are the four main fibers that have been widely used in engineering applications [9]. However, some carcinogens in asbestos fiber are volatile during application, so this fiber has been abandoned in many countries. Comparatively, basalt fiber is an eco-friendly material and possesses good performance in thermostability, physical, and mechanical properties, and chemical stability [5]. Although some different fibers have been utilized and their good performance has been verified, comparative research between different fibers and fiber-reinforced asphalt binder in order to select the favorable fiber type are scarce.

In addition, the influence of fiber size on fiber-reinforced asphalt mixtures has been observed and some related research was conducted, where the size mainly included the diameter and length [10,11]. Some researchers have shown that the effect of fiber length on mixture properties is more significant than that of fiber diameter. Moreover, considering that the diameters of fibers are determined by complex technologies and are generally provided by producers, the influence of fiber length on mixture properties has been focused upon [12]. Fiber with an excessively long length may cause a negative effect on stability and reinforcement, while fiber with a relatively shorter length will lead to the agglomeration phenomenon, caused by the nonuniform distribution. There is scarce research on the effect of fiber length on mixture performance. Therefore, a favorable fiber length is required for the fiber-reinforced asphalt mixture with a good performance.

Moreover, the optimal fiber content should be determined as well, as related experiments were conducted and the results showed that the fiber content significant influenced the performance of the asphalt binder and asphalt mixture. As for the fiber-reinforced asphalt binder, the fundamental properties, e.g., penetration, softening point, ductility, viscosity [13], asphalt film thickness, richness modulus [14], and rheological properties, e.g., dynamic shear modulus [15], creep stiffness and creep rate [16] will be changed with the addition of fiber. As for fiber-reinforced asphalt mixture, the mechanical properties, e.g., indirect tensile strength [17,18], engineering properties, such as high temperature rutting performance, low temperature cracking resistance performance, and moisture susceptibility performance [19,20,21] are also changed with the addition of different fiber contents. Therefore, the influence of fiber content on asphalt mixture should be evaluated and the optimal fiber content should be recommended for a practical application.

In this study, the fiber type, fiber length and fiber content as three key parameters that significantly influence the fiber-reinforced asphalt mixture performance were focused and investigated systematically. Experiments on fibers, fiber-reinforced asphalt binder, and fiber-reinforced asphalt mixture were conducted. Specifically, three kinds of fibers, namely basalt fiber, polyester fiber, and lignin fiber were selected, and fiber property tests include physical and mechanical properties test, oven heating test, water absorption test, and asphalt binder absorption test were conducted to determine the favorable fiber type, shown in Section 3.1.1. Furthermore, three kinds of fiber-reinforced binder were prepared and then penetration test, softening point test, and ductility test were conducted to determine the favorable fiber type further, shown in Section 3.1.2. After the favorable fiber type was determined, fiber-reinforced asphalt binders with different fiber lengths were prepared, and the mesh-basket draindown test and cone penetration test were conducted to determine the favorable fiber length, shown in Section 3.2. Subsequently, Marshall test for the fiber-reinforced asphalt mixture was conducted under favorable fiber type and length to determine the optimum asphalt binder content under different fiber contents, shown in Section 3.3.1. Finally, the engineering properties including high temperature rutting test, low temperature bending test, and moisture susceptibility test were verified and the optimum fiber content was determined as well, shown in Section 3.3.2, Section 3.3.3, and Section 3.3.4.

## 2. Materials and Mixing

### 2.1. Raw Materials

#### 2.1.1. Asphalt Binder

90# penetration grade asphalt binder, provided by Panjin Northern Asphalt Co., Ltd., located in Liaoning province of China, was selected in this study, and the physical properties of asphalt binder are shown in Table 1 following American Society of Testing Materials (ASTM) standards.

#### 2.1.2. Fibers

**Figure 1:**
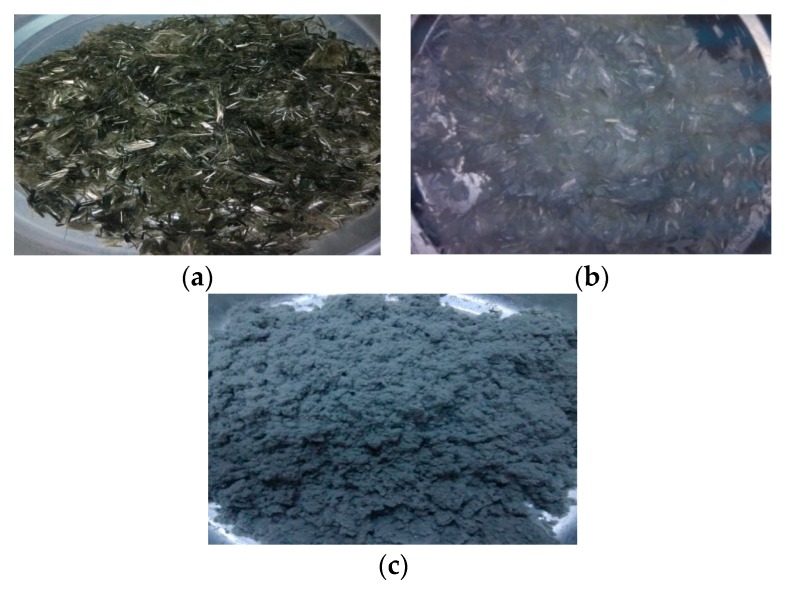
Basic appearance of three kinds of fibers: (**a**) Basalt fiber; (**b**) Polyester fiber; (**c**) Lignin fiber.

Basalt fiber was produced by Shijin Basalt Fiber Co., Ltd., located in Zhejiang of China, and the basic appearance is shown in Figure 1a. The properties of basalt fiber are presented in Table 2. Polyester fiber was produced by Changzhou Tianyi Engineering Fiber Co., Ltd., located in Jiangsu of China, and the basic appearance and its properties are presented in Figure 1b and Table 3, respectively. Lignin fiber was provided by Langfang Hexiang Building Materials Co., Ltd., located in Hebei province of China, where the basic appearance and properties of this kind of fiber are shown in Figure 1c and Table 4, respectively. All properties for these three kinds of fibers were provided by manufacturers.

#### 2.1.3. Aggregates and Mineral Fillers

Aggregates used in this study, including coarse aggregate and fine aggregate, were the crushed basalt, which was provided by Dengzhou Stone Materials Factory, located in Henan province of China. Mineral fillers used in this study were produced by the crushed limestone from Dengzhou Stone Materials Factory as well. All of the aggregates and fillers used in this study meet the specification requirements of Technical Specification for Construction of Highway Asphalt Pavements (JTG F40-2004) [22].

### 2.2. Mixing

#### 2.2.1. Preparation of Fiber-Reinforced Asphalt Binder

The fiber-reinforced asphalt binder was prepared by mixing asphalt binder and fibers together. Firstly, the asphalt binder was heated to nearly 150 °C, and fibers were heated to nearly 105 °C to keep them dry sufficiently. Afterwards, fibers with different contents were added into heated asphalt binder to finish the preparation of fiber-reinforced asphalt binder, where the fibers were added steadily and stirred at high speed (generally larger than 500 revolutions per minute) simultaneously by a blender in order to ensure the dispersion uniformity of fibers.

#### 2.2.2. Selection of Mixture Gradation

**Figure 2:**
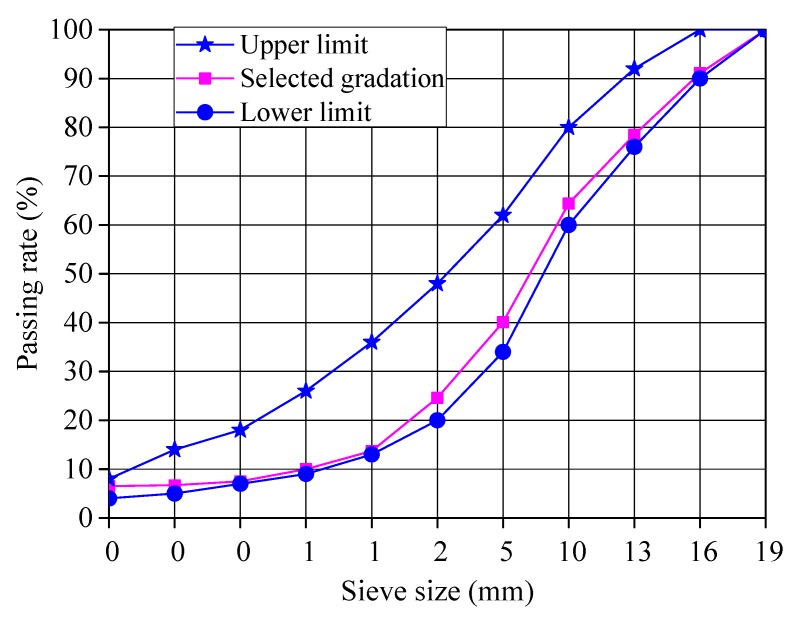
Gradation composition curve of AC-16 asphalt mixture.

The aggregate gradation composition of AC-16 asphalt mixture was selected in this study. According to the gradation requirement, the selected gradation composition curve of AC-16 asphalt mixture was shown in Figure 2.

## 3. Testing Methods

**Figure 3:**
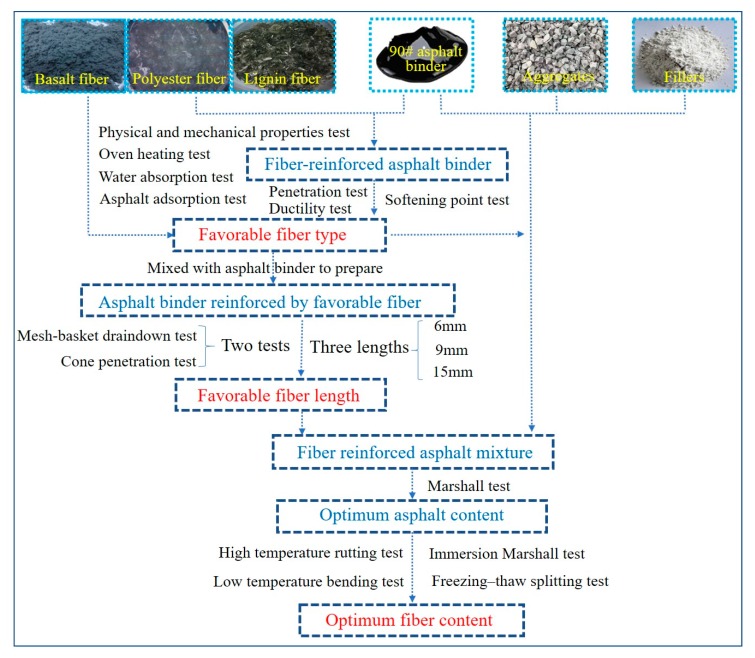
Flow chart of testing methods.

Experiments on the prepared fibers, fiber-reinforced asphalt binder, and fiber-reinforced asphalt mixture were conducted to determine the favorable parameters in terms of fiber type, fiber length, and fiber content. In this study, the flow chart of testing methods is summarized in Figure 3.

### 3.1. Determination of Favorable Fiber Type

In order to determine the favorable fiber type, three kinds of fibers and three kinds of fiber-reinforced asphalt binder were prepared. The comparison among these three kinds of fibers was conducted in term of their basic properties to recommend the favorable fiber type, where physical and mechanical properties test, oven heating test, water absorption test, and asphalt binder adsorption capacity test were conducted. As for the three kinds of fiber-reinforced asphalt binder, their physical performance was measured, including penetration test, softening point test, and ductility test, where different contents (2 wt.%, 4 wt.%, 6 wt.%, 8 wt.%) for each fiber were selected.

#### 3.1.1. Favorable Selection of Fiber Type Based on Fibers Property

1. Physical and mechanical properties test

The basic physical properties, including diameter, length, and density were measured and color of each fiber was observed visually. Moreover, the mechanical property, namely fracture strength test was also conducted to compare the ultimate strength under the traction.

2. Oven heating test

The oven heating test as a sample test is used to evaluate the thermostability of fibers, which was conducted to verify the compacity of fibers to maintain their original properties at high temperature. In this study, three kinds of fibers (10 g of each fiber) were placed separately in the oven with a constant temperature of 210 °C. After heating for 5 h, the samples were taken out. The loss weight could then be weighed and the appearance change could also be observed. During the heating process, three repeat samples were prepared and tested for each fiber type.

3. Water absorption test

Low water absorption is key to ensure that fiber-reinforced asphalt mixture is protected from moisture damage during the service stage. The water absorption test was conducted for each fiber. The basic procedures were described as follows: each of fiber sample with 11 g weight was prepared and placed in a dry beaker. Subsequently, the prepared samples were exposed in air in a curing chamber for 2 days, where the relative humidity was set at 90% and the temperature was set as 20 °C. Afterwards, the total weight for each sample was measured and the absorbed water could be calculated. It should be noted that three repeat samples were tested for each fiber.

4. Asphalt binder adsorption test

A good performance in binder absorption property is key to ensure the good compatibility between fibers and asphalt binder, and it can also prevent the bleeding of fiber-reinforced asphalt mixtures in summer. The asphalt binder adsorption test was conducted by a JJYMX-1 fiber binder absorption measurement apparatus. The procedures were as follows: (1) three kinds of dry fibers were prepared in a vessel with 5 g weight for each of them (where the mass of fibers is *m*_1_); (2) an asphalt binder with 100 g weight was poured into the vessel and was steadily stirred by a glass rod to mix them together for 15 min and then maintained for 5 min; (3) the mass of the sample sieve was weighed as *m*_2_ and was stalled in the binder absorption measurement apparatus; (4) the prepared mix was placed on sample sieve; (5) the apparatus was initiated and worked normally for 10 min and then stopped immediately; (6) the sample sieve was taken down and was weighted as *m*_3_ (where the mass of *m*_3_ included the weight of sieve and fibers with adsorption of asphalt binder). According to the procedures, the adsorption ratio φ of fiber to binder can be calculated as:

(1) $$\varphi =\frac{m_{3}-m_{2}-m_{1}}{m_{1}}$$

#### 3.1.2. Favorable Selection of Fiber Type Based on Fiber-Reinforced Asphalt Binder Performance

Three kinds of fiber-reinforced asphalt binder were prepared according to Section 2.2.1. Afterwards, the penetration test, the softening point test, and the ductility test were conducted according to ASTM D5-97 standard [23], ASTM D36-95 standard [24], and ASTM D113-99 standards [25], respectively.

According to the test results, the favorable fiber type can be deduced. Afterwards, the favorable fiber type will be selected eventually by combining two categories of results based on fiber properties and fiber-reinforced asphalt binder performance.

### 3.2. Determination of Favorable Fiber Length

After the favorable fiber type was determined, the favorable length needed to be determined for good performance after adding fiber into the asphalt binder and then the asphalt mixture. In this study, two experiments were conducted under three different lengths (6, 9, and 15 mm) to determine the favorable length of the favorable fiber based on the prepared fiber-reinforced asphalt binder, namely, the mesh-basket draindown test and cone penetration test.

#### 3.2.1. Mesh-Basket Draindown Test

**Figure 4:**
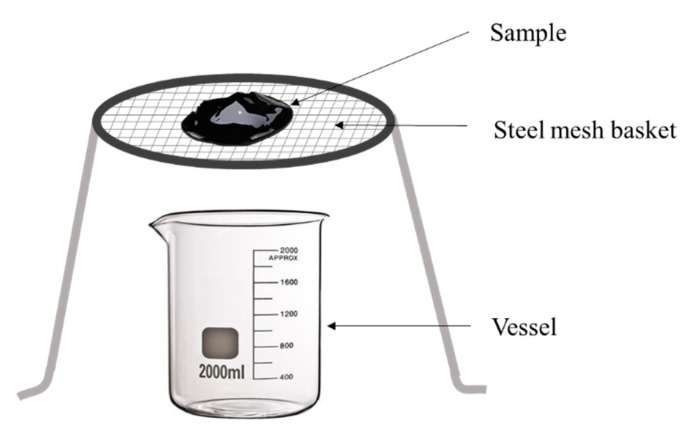
Schematic diagram of mesh-basket draindown test.

The mesh-basket draindown test was initially proposed by Tongji university and has been used widely as an effective method to determine the adsorption of fibers to asphalt binder and the stabilization of fiber-reinforced asphalt binder [5]. The schematic diagram of this device is shown in Figure 4, mainly consisting of sample, steel mesh basket, and a vessel for dropping fiber-reinforced asphalt binder collection. During the process, the favorable fiber type determined from Section 3.1 with 10 g (10 wt.%) and 100 g asphalt binder was selected and mixed together according to procedures described in Section 2.2, where different lengths of fibers were selected to investigate the influence of fiber length on fiber-reinforced asphalt binder properties. After the sample was mixed and prepared, it was placed on a stainless-steel mesh basket (where the size of sieve was set as 0.25 mm), and then the prepared sample with the mesh basket was placed in the oven at 25 °C for 1 h. After the sample was completely cooled, the weight of sample could be measured. Afterwards, three repeat samples with mesh baskets were heated at 130 °C, 160 °C, 190 °C for 1h, respectively. During this heating process, the separated weight of the asphalt binder from the prepared sample could be measured every 30 min, thus the mass loss rate of the sample could be calculated by the following equation:

(2) $$\delta =\frac{m_{L}}{m_{0}}$$

where δ is mass loss rate, *m_L_* is average mass loss of the measurement after 30 and 60 min heating, and *m*_0_ is the initial mass before heated.

To ensure the accuracy of the measured results, the tests with three same samples were conducted under each temperature and the average values for the fiber-reinforced asphalt binder could be calculated. The mass loss rate can be used to reflect the adsorption of fibers to asphalt binder. A good performance in adsorption properties can be achieved with a relatively low mass loss rate.

#### 3.2.2. Cone Penetration Test

**Figure 5:**
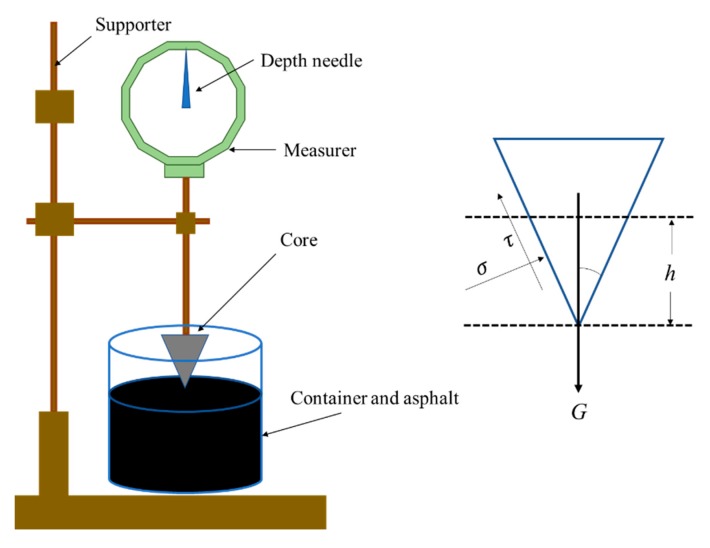
Schematic diagram of cone penetration test.

The cone penetration test was designed to evaluate the resistance to flow and shear for the fiber-reinforced asphalt binder. The schematic diagram of the cone penetration test was depicted as shown in Figure 5 [21]. The procedures of the test include: an asphalt binder of 500 g and a type of fiber of 50 g (10 wt.%) with different lengths were mixed together according to the steps in Section 2.2. Afterwards, the prepared fiber-reinforced asphalt binder sample was poured into an iron vessel steadily with the aid of a funnel to form a uniform distributed mix in the vessel. Subsequently, the sample in the vessel was maintained at room temperature for 50 min until it cooled, and then it was immersed into water with a constant temperature of 25 °C for about 1 h. Later, the sample was removed from water and put beneath the cone. The iron cone weighing 500 g was released from the sample surface. Afterwards, the cone would penetrate into the sample gradually until it reached a stable state for the visco-elastic-plastic property of asphalt binder. During this sink process, the sink depth of cone was recorded. According to the force balance theory (the right part of Figure 4), the shear stress *τ* (kPa) of the fiber-reinforced asphalt binder at the tangential direction to the cone surface can be calculated as follows:

(3) $$\tau =\frac{W\cos^{2}\left(\frac{\alpha }{2}\right)}{\pi h^{2}\tan\left(\frac{\alpha }{2}\right)}$$

where *W* is the weight of the cone (5 kN), *h* is the sink depth of the cone (m), and α is the cone angle (30°). In order to obtain a reliable result, three repeat samples were prepared for the determination of the favorable fiber length. Obviously, a large shear stress τ is favored for fiber-reinforced asphalt binder.

Based on results from mesh-basket draindown test and cone penetration test, the favorable fiber length can be determined by comparing the adsorption of fiber to asphalt binder and shear stress of fiber-reinforced asphalt binder with different fiber lengths.

### 3.3. Determination of Optimum Fiber Content and Verification of Engineering Properties

After the favorable type and the favorable length of the fiber was determined, the gradation of AC-16 was selected to prepare the fiber modified asphalt mixture. Different contents of fiber were selected, and then the Marshall test was conducted to determine the optimum asphalt binder content with different fiber contents. Afterwards, the engineering properties were investigated and verified, including the high temperature rutting test, low temperature bending test, and moisture susceptibility test. In this study, three repeat specimens were prepared for each engineering property verification.

#### 3.3.1. Determination of Optimum Asphalt Binder Content

**Figure 6:**
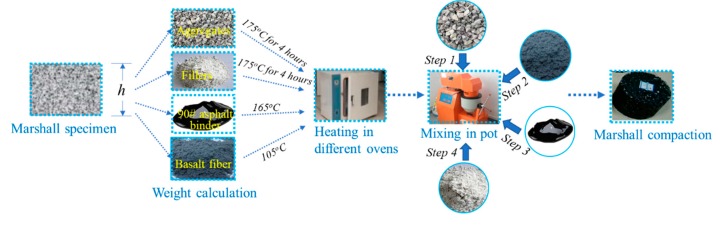
Graphical procedures of Marshall specimen preparation.

The optimum asphalt binder contents for fiber-reinforced asphalt mixture with different fiber contents were determined by the Marshall method. In this study, six fiber contents were selected, namely 0 wt.% (as control group), 0.2 wt.%, 0.3 wt.%, 0.4 wt.%, 0.5wt.%, and 0.6 wt.%. Before the test, the Marshall specimens needed to be prepared. The procedures of this were as follows: (1) The total quality of asphalt mixtures was determined by the height of the Marshall specimens, and then the contents of asphalt binder, fibers, and each component for aggregates was determined; (2) The aggregates and fillers were put in the oven under 175 °C for 4 h, and the fibers and asphalt binder were heated to 105 °C and 165 °C, respectively; (3) Aggregates were poured into mixing pot at 175 °C, and then fibers were put into aggregates and stirred for 30 s to keep a good homogeneity with aggregates, followed by the addition of the asphalt binder and stirring for 60 s, and the mineral fillers were finally added and stirred for 90 s. (4) The prepared loose mixtures were put into a standard Marshall mould and underwent the Marshall compaction procedure, namely double-sided compaction for 75 blows, and then they were cooled at room temperature for 24 h for demoulding. The graphical procedures of Marshall specimen preparation are shown in Figure 6.

After the Marshall specimens were prepared, the Marshall test could be conducted according to the ASTM D1559 standard [26]. As for the contents of each fiber, five different asphalt binder contents, namely asphalt binder contents vary from 3.4%–5% with 0.4% as interval, were selected to conduct the Marshall test. Afterwards, the physical and mechanical parameters could be obtained to calculate the optimum asphalt binder content under different fiber contents.

#### 3.3.2. High Temperature Rutting Test

**Figure 7:**
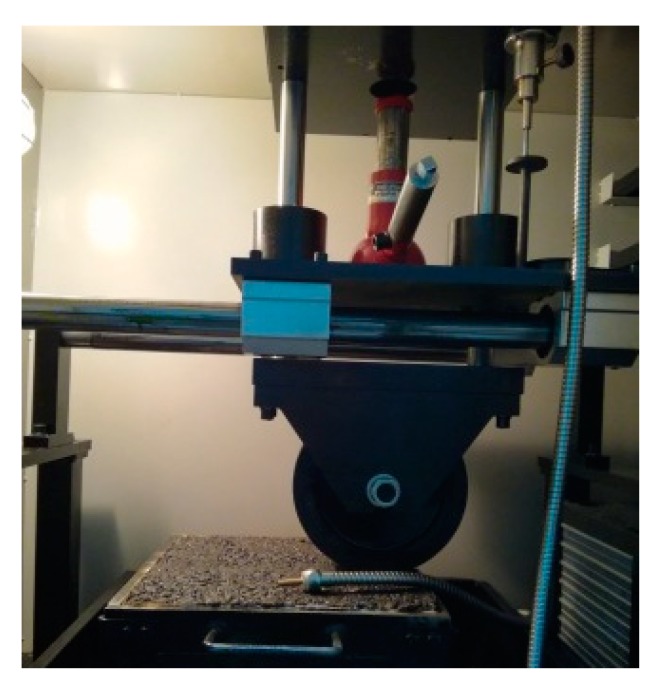
Rutting test process.

Asphalt pavement is prone to accumulate permanent deformation under repeated vehicle moving loads, especially in high temperature environments in summer, causing rutting. In order to obtain a good performance in resisting permanent deformation, the adequate high temperature performance is required for the asphalt mixture, as well as the fiber-reinforced asphalt mixture. Generally, the wheel tracking test has been widely used to evaluate the high temperature stability properties of different asphalt mixtures [27]. The rutting test process in this study was shown in Figure 7.

During the test process, the square slab specimens (300 mm × 300 mm × 50 mm) were prepared under the optimum asphalt binder contents obtained in Section 3.3.1, the obtained aggregates gradation in Section 2.2.2, and the favorable fiber type and length in Section 3.1 and Section 3.2 according to the Marshall test requirement. During the rutting test process, the specimens were first put in a room with a constant temperature of 60 ± 0.5 °C for 24 h. Subsequently, the samples were loaded with a solid rubber tire wheel (the contact stress was 0.7 MPa), where the traveling distance was 230 ± 10 mm, and the running speed was 42 cycles/min. The whole test process, including the loading and rolling process, was carried out for 60 min. After the test was completed, the dynamic stability (*DS*) could be calculated according the recorded data during the testing process, and the equation is shown as follows:

(4) $$DS=\frac{15\times 42}{d_{60}-d_{45}}$$

where *DS* is the dynamic stability of specimens (cycles/mm); 15 is the time difference between 60 min and 45 min (min); 42 is the running speed (cycles/min); *d*_60_ and *d*_45_ is the tracking depth (mm) of specimen surface at 60 min and 45 min, respectively.

#### 3.3.3. Low Temperature Bending Test

**Figure 8:**
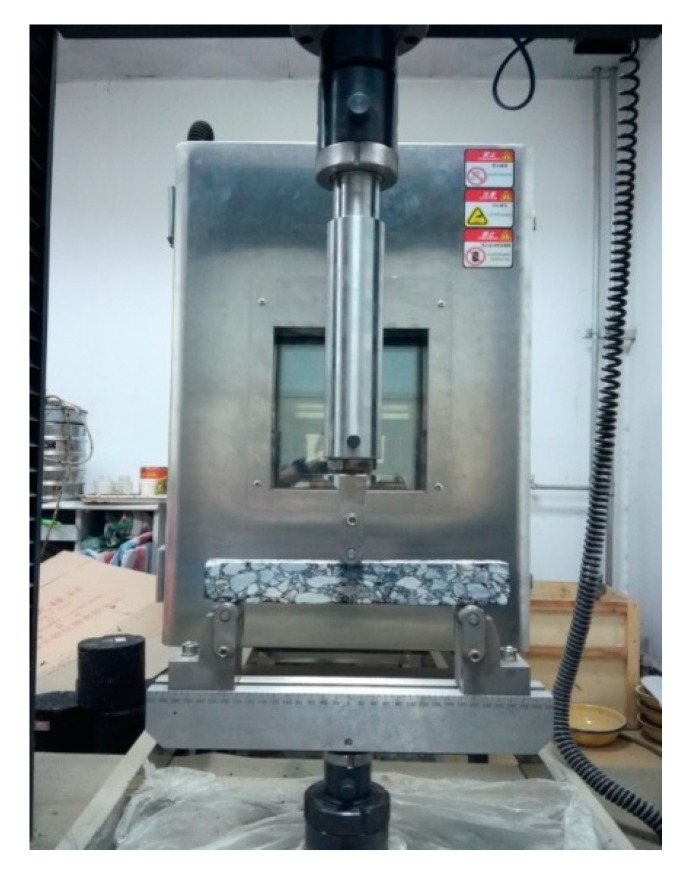
Low temperature bending test process.

In order to have good cracking resistance for fiber-reinforced asphalt mixture under low temperature, especially in winter, the bending test, as a widely used method, was conducted to evaluate the performance of asphalt mixtures with different fiber contents [27]. The low temperature bending test is shown in Figure 8.

The specimens used for the bending test were prepared initially with a size of 40 mm × 40 mm × 240 mm. Afterwards, the specimens were maintained in an environmental chamber at −10 °C for 6 h. Then, the specimens were placed on the closed-loop controlled servohydraulic MTS 810 material test system (MTS Systems Corporation, Minneapolis, USA). After that, the three-point flexural loading method was adopted, where the span length was set as 200 mm, the loading rate was set as 20 mm/min, and the test temperature was set as −10 °C. During the test process, the maximum load and the mid-span deflection at the specimen failure could be recorded. Thus, the maximum tensile stress and the maximum strain at mid-span can be calculated by the following equations:

(5) $$R_{B}=\frac{3LP_{B}}{2bh^{2}};\varepsilon_{B}=\frac{6hd}{L^{2}}$$

where *R_B_* is the maximum tensile stress (MPa); $\varepsilon_{B}$ is the maximum strain, *L* is the span length (mm); *P_B_* is the maximum load at failure (N); *b* is the width of the cross section (mm); *h* is the height of the cross section (mm); *d* is the mid-span deflection at the specimen failure (mm).

#### 3.3.4. Moisture Susceptibility Test

Asphalt as a binder is the key component in asphalt mixture to connect the aggregates together to form a framework. However, the interface between the asphalt binder and aggregate is prone to damage under moisture, as the affinity of water to aggregate is better than binder to aggregate. In order to have a good performance under moisture, the fiber-reinforced asphalt mixture’s performance of moisture susceptibility should be verified. The immersion Marshall test and freeze–thaw splitting test, two widely used methods, were conducted in this study to investigate the performance of moisture susceptibility in fiber-reinforced asphalt mixtures with different contents.

1. Immersion Marshall test

During the immersion Marshall test (T 0709-2011) [27], eight specimens of fiber-reinforced asphalt mixture with different fiber contents were prepared and then were divided into two groups, one group (namely four specimens for each fiber content) was immersed into water for 30 min at 60 °C, another was immersed into water for 48 h at 60 °C. Afterwards, the Marshall test was conducted for each specimen, and the residual stability (*MS*_0_) was calculated according to the Marshall stability values for former and latter group.

2. Freeze–thaw splitting test

Similar to immersion Marshall test, the freeze–thaw cycling test was conducted for prepared Marshall specimens in the two groups, where each group has four specimens (AASHTO T-283) [28]. One group was be maintained at room temperature during the test preparation. Another group was vacuumed in water for 15 min, and then was put in a water bath (where the water temperature was set to −18 °C) for 16 h. Subsequently, the frozen group was thawed in the water bath at 60 °C for 24 h. Afterwards, all specimens, including the two groups, were put in water bath for 2 h at 25 °C. Finally, the indirect tensile test (splitting test) was conducted with a loading rate of 50 mm/min for all specimens and the indirect tensile strength could be determined. The moisture susceptibility can be evaluated by using the tensile strength ratio (*TSR*), which can be calculated based on the equation as following:

(6) $$TSR=\frac{R_{T2}}{R_{T1}}\times 100$$

where *TSR* is the tensile strength ratio (%); *R*_*T*2_ is the average tensile strength of specimens for unconditioned group (MPa); *R*_*T*1_ is the average tensile strength of specimens for the group with a freeze–thaw cycle (MPa).

According to the results of the engineering properties for fiber-reinforced asphalt mixtures with different fiber contents, the optimum fiber content was obtained and the engineering properties will be verified in the following.

## 4. Result and Discussion

### 4.1. Favorable Fiber Type

#### 4.1.1. Fiber Property

1. Physical and mechanical property

The physical and mechanical properties for three kinds of fibers are measured as shown in Table 5.

As can be seen from Table 5, the mechanical property of the basalt fiber is relatively optimal, and the fracture strength is much larger than that of the polyester fiber and the lignin fiber. Moreover, the density of basalt fiber is nearly twice the density of polyester fiber and lignin fiber.

2. Oven heating test

The oven heating test was conducted to evaluate the thermostability for three kinds of fibers, and the average results are shown in Table 6.

Table 6 shows that the basalt fiber has the lowest mass loss after oven heating, which means it possesses the highest thermostability among the three fibers. The thermostability for these three fibers follows in this order: basalt fiber > polyester fiber > lignin fiber. In addition, the color change for the lignin fiber is apparent, which changed from gray to yellow.

3. Water absorption test

Adhering to Section 3.1.1, the water absorption results for three kinds of fiber are shown in Table 7.

From Table 7, the water absorption ratios for the basalt fiber, polyester fiber, and lignin fiber, are 0.8 wt.%, 3.7 wt.%, and 24.4 wt.%, respectively. Clearly, the water adsorption for lignin fiber is greatest, which adsorbs much more water than other two fibers after two days in the moisture condition, where the water adsorption can be detected even by the finger. Similarly to the results of the physical test and the oven heating test, the water adsorption performance for the basalt fiber is relatively optimal.

4. Asphalt binder adsorption test

The asphalt binder adsorption test for three kinds of fibers was conducted based on a JJYMX-1 asphalt binder absorption measurement apparatus, and the results are shown in Table 8.

It can be observed from Table 8 that lignin fiber has the most optimal performance in asphalt binder adsorption property relatively, which can be attributed to a large number of voids in this fiber, which is sourced from plants. The asphalt binder adsorption ratio for basalt fiber is 62%, which meets the specification requirement of the asphalt binder adsorption.

Combined with the fiber properties obtained above, the basalt fiber was selected as the most favorable type.

#### 4.1.2. Fiber-Reinforced Asphalt Binder Performance

1. Penetration test

**Figure 9:**
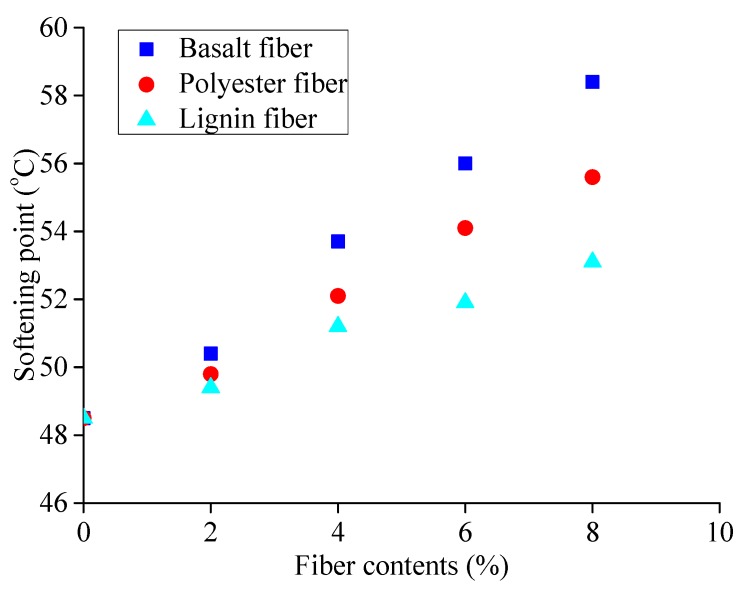
Penetration test results of three kinds of fiber-reinforced asphalt binder, where the testing temperature was set to 25 °C.

The penetration test results for three kinds of fiber-reinforced asphalt binder with different fiber contents are depicted in Figure 9.

From Figure 7, the penetration values decrease with the increase of fiber contents three kinds of fiber-reinforced asphalt binder. This is because the fiber network, formed by the addition of fibers, will restrict the flowability of asphalt binder, thereby increasing the consistency of the asphalt binder and reducing the temperature sensitivity of the binder. Compared with the polyester fiber and lignin fiber, the basalt fiber has the greatest impact on the penetration of the asphalt binder, that is, it possesses more significant viscosifying effect.

2. Softening point test

**Figure 10:**
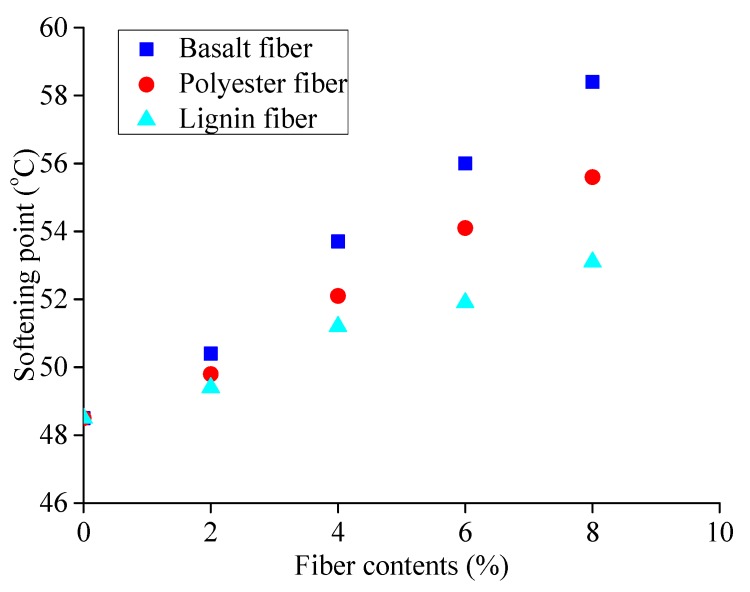
Softening point test results for three kinds of fiber-reinforced asphalt binder.

The test results of the softening point for three kinds of fiber-reinforced asphalt binder with different fiber contents are shown in Figure 10.

It can be seen from Figure 10 that the softening point increases linearly with the fiber contents in three kinds of fiber-reinforced asphalt binder, which means the high temperature stability for the asphalt binder was improved by the addition of fibers. The high temperature improvement effect of the fibers on the asphalt binder follows this order: basalt fiber > polyester fiber > lignin fiber.

3. Ductility test

**Figure 11:**
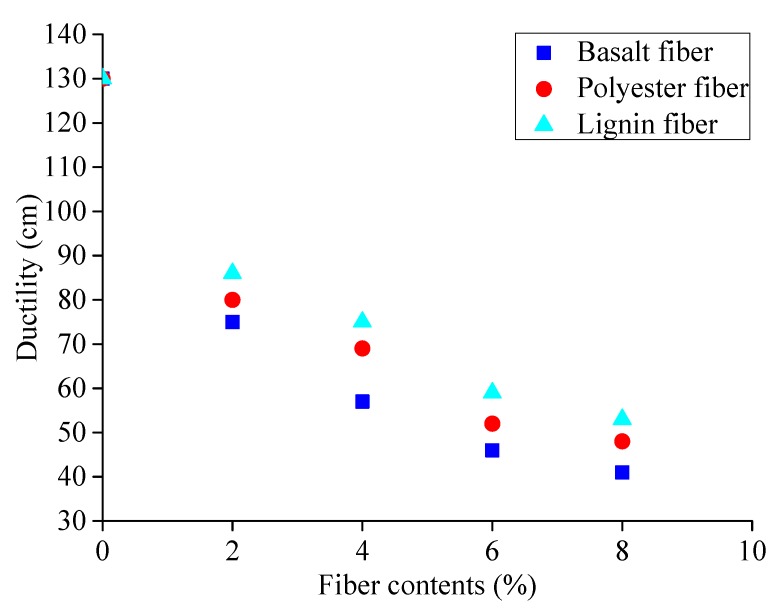
Ductility test results for three kinds of fiber-reinforced asphalt binder, where the testing temperature was set to 15 °C.

The ductility test results for three kinds of fiber-reinforced asphalt binder with different fiber contents at 15 °C are shown in Figure 11.

Figure 9 shows that the ductility of three fiber-reinforced asphalt binders sharply decreases with the fiber contents, which means that the proper contents for each fiber should be selected because the excessive content of fibers leads to a poor low-temperature performance.

After comparison among the three kinds of fiber-reinforced asphalt binders in terms of fiber properties and fiber-reinforced asphalt binder performance, the basalt fiber was selected as the favorable fiber type.

### 4.2. Favorable Fiber Length

Based on results from Section 4.1, basalt fiber was selected. In this section, the mesh-basket draindown test and cone penetration test were conducted for basalt fiber-reinforced asphalt binder with different lengths to determine the favorable fiber length.

#### 4.2.1. Mesh-Basket Draindown Test

**Figure 12:**
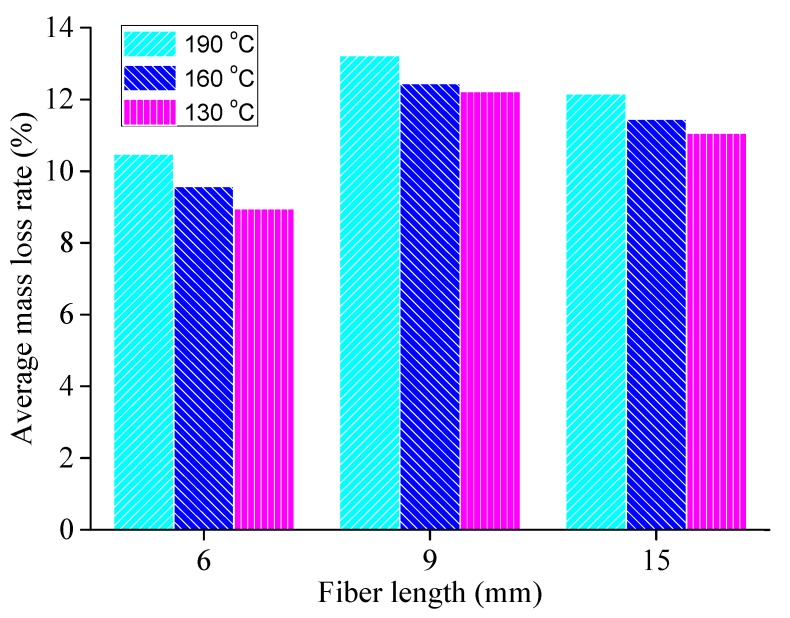
Mass loss rate for fiber-reinforced asphalt binder at different temperatures.

According to the procedures described in Section 3.2.1, basalt fibers with diameter of 17 μm and of three lengths (6 mm, 9 mm and 15 mm) were selected. The mesh-basket draindown test was conducted and the average mass loss rate for the fiber-reinforced asphalt binder at different temperatures was calculated as shown in Figure 12.

As can be seen from Figure 12, the average mass loss rate of the fiber-reinforced asphalt binder with three fiber lengths decreases with the decrease of temperature, which is consistent with the common understanding of mass loss. The mass loss ratio for the fiber-reinforced asphalt binder with different fiber lengths follows this order: 9 mm length fiber > 15 mm length fiber > 6 mm length fiber. Therefore, the adsorption property of the fiber-reinforced asphalt binder for basalt fiber with 6 mm length is most favorable.

#### 4.2.2. Cone Penetration Test

**Figure 13:**
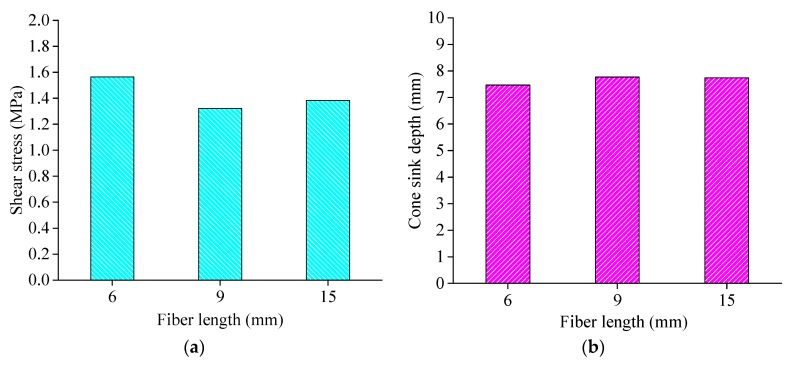
Shear stress and cone sink depth for fiber-reinforced asphalt binder with different fiber lengths: (**a**) Shear stress; (**b**) Cone sink depth. The testing temperature was set to 25 °C.

The cone penetration test was conducted for the basalt fiber-reinforced asphalt binder with three fiber lengths, where the fiber lengths were 6 mm, 9 mm, and 15 mm, respectively. The shear stress and cone sink depth were recorded and the average value was calculated as shown in Figure 13.

Figure 13 shows that the shear stress for the fiber-reinforced asphalt binder with different fiber lengths presents the same order as the adsorption property adopted by the mesh-basket draindown test. The shear stress of the fiber-reinforced asphalt binder for basalt fiber with 6 mm length is optimal, relatively.

Combined with the results from Section 4.2.1 and Section 4.2.2, the basalt fiber with 6 mm length was selected as the favorable length.

### 4.3. Optimum Fiber Content and Engineering Properties

#### 4.3.1. Optimum Asphalt Binder Content

**Figure 14:**
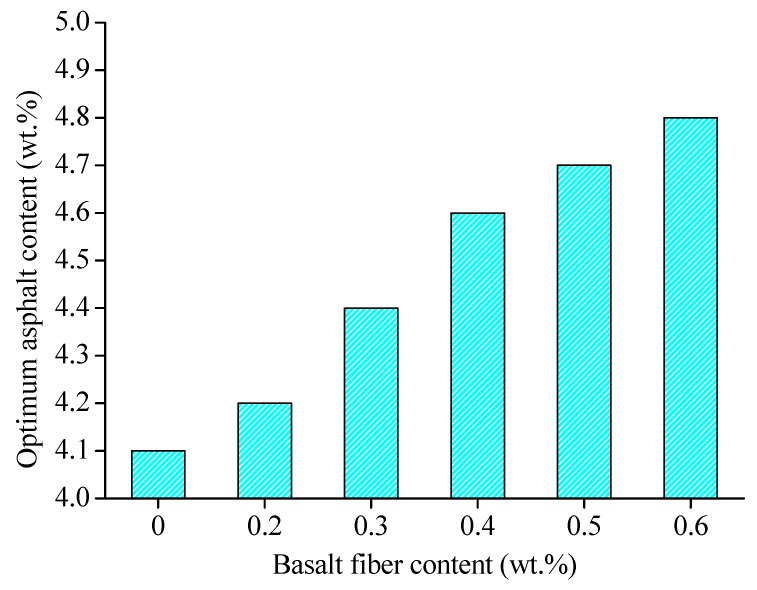
Optimum asphalt binder contents for different fiber contents.

According to experimental planning in Section 3.3.1, the Marshall test was conducted for the preparation of Marshall specimens with different fiber contents. After the Marshall test was completed, the optimum asphalt binder contents of different fiber contents could be determined according to the procedures described in the Marshall test. The calculated results were obtained as shown in Figure 14.

Figure 14 shows that the optimum asphalt binder content will increase with the fiber content. This may be caused by adsorption of fiber to asphalt binder. After the addition of fiber, a part of the mass of asphalt binder is absorbed by basalt fiber, which means that asphalt mixture needs more binder to supply this lost mass to achieve the best performance. However, considering that the addition of fiber is a complex process, the more reasonable explanation needs to be investigated further.

#### 4.3.2. High Temperature Rutting Test

**Figure 15:**
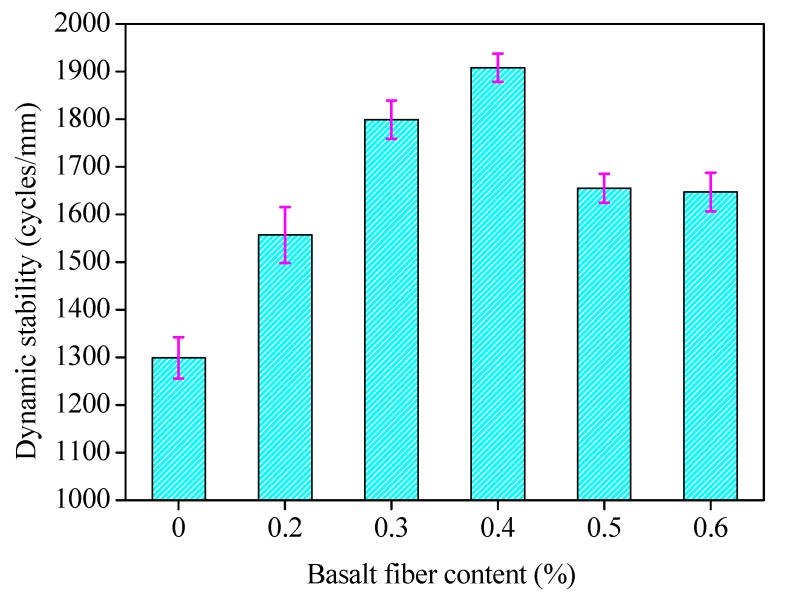
Dynamic stability for fiber-reinforced asphalt mixture with different fiber contents, where the testing temperature was set to 60 °C.

The high temperature rutting test was conducted under the optimum asphalt binder contents for fiber-reinforced asphalt mixtures with six different fiber contents. The test results of the dynamic stability for mixture with different fiber contents are shown in Figure 15, where the error bars represent the standard variations of testing results.

As can be seen in Figure 15, the high temperature stability of asphalt mixture was enhanced significantly with the increase of fiber content. The dynamic stability of the mixture increased with the basalt fiber content at first, and decreased later, as the dynamic stability reached a peak value under the 0.4 wt.% fiber content. This may be because the fiber can be fully dispersed in the asphalt mixture when the basalt fiber content is low. The high temperature deformation resistance of the asphalt mixture can be improved by basalt fiber to increase the viscosity of asphalt binder and reinforce asphalt mixture. With the increase of fiber content, the high temperature stability of asphalt mixture increases rapidly before the fiber content in asphalt mixture reaches the optimal content. When the fiber content exceeds this optimal value, the appearance of agglomeration phenomena in fibers, caused by uneven dispersion in the asphalt mixture and an excessively adsorptive asphalt binder, will reduce the high temperature stability. Therefore, the optimum fiber content is 0.4 wt.% in terms of high temperature performance.

#### 4.3.3. Low Temperature Bending Test

**Figure 16:**
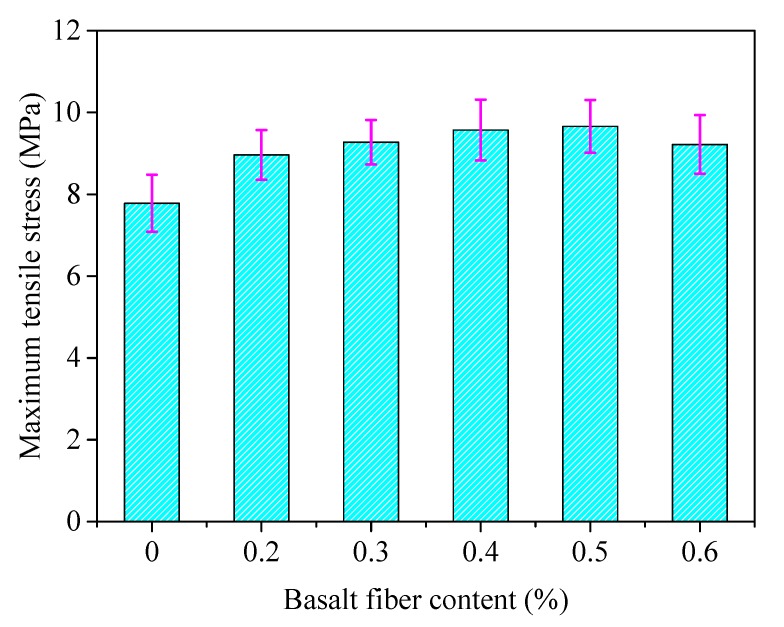
Maximum tensile stress of basalt reinforced asphalt mixture with different fiber contents, where the testing temperature was set to −10 °C.

**Figure 17:**
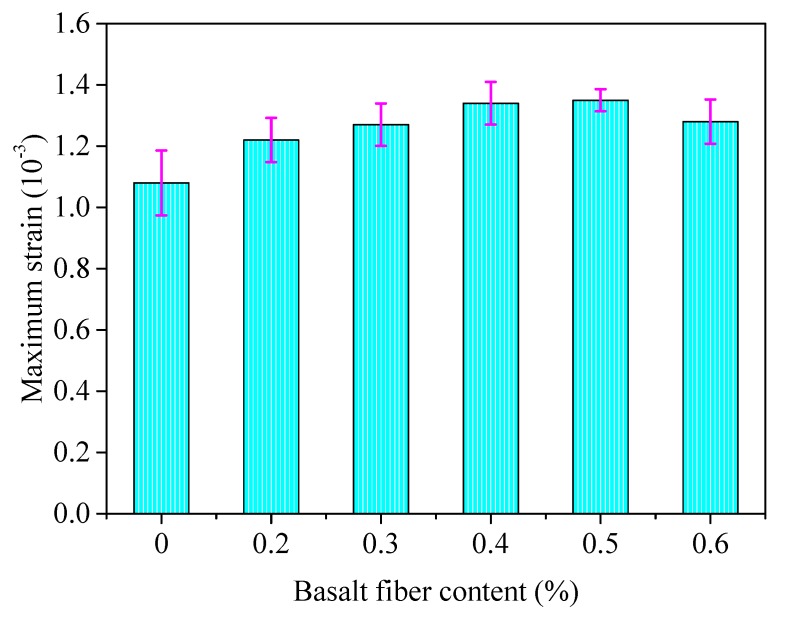
Maximum strain of basalt reinforced asphalt mixture with different fiber contents, where the testing temperature was set to −10 °C.

The same components of asphalt mixture were selected as described in Section 4.3.1, and then the low temperature bending test was conducted. The maximum tensile stress and the maximum strain at mid-span could be calculated after the test, and the results are shown in Figure 16 and Figure 17, where the error bars represent the standard variations of testing results.

From Figure 16 and Figure 17, the low temperature cracking resistance of basalt fiber-reinforced asphalt mixture was improved slightly with the addition of basalt fiber compared to the unreinforced specimens. The maximum tensile stress and strain of basalt fiber-reinforced asphalt mixture increased initially and decreased afterwards with the increase of fiber contents, where the maximum tensile stress and strain reach to a maximum value when the fiber content is approximately 0.4–0.5 wt.%.

The reason for this could be explained, as basalt fiber with a relative low content can enhance the mechanical property of asphalt mixture at low temperature by distributing uniformly in asphalt mixture and forming a network structure. However, the agglomeration phenomena appeared for a relatively high fiber content, which impaired the mechanical strength at low temperatures, but the performance was still better than the control group. Further studies can be conducted to investigate the limited fiber content, where the performance is worse than the virgin asphalt mixture.

#### 4.3.4. Moisture Susceptibility Test

1. Immersion Marshall test

**Figure 18:**
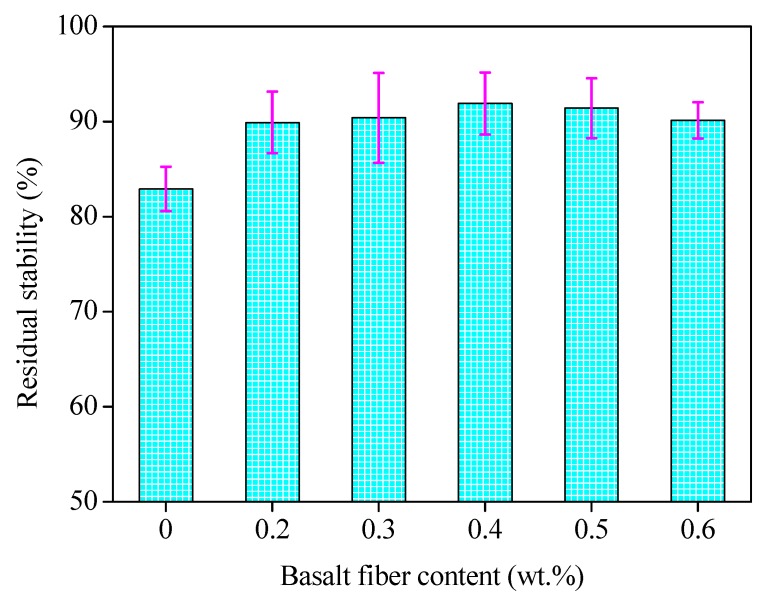
Residual stability of basalt reinforced asphalt mixture with different fiber contents.

The immersion Marshall test was conducted based on prepared Marshall specimens, and the results of the residual stability (*MS*_0_) are shown in Figure 18, where the error bars represent the standard variations of testing results.

It can be seen in Figure 18 that the residual stability values for all mixture specimens are larger than 80%, satisfying the specification requirements. The residual stability increased initially and then decreased later, where a peak value appeared with the 0.4 wt.% basalt fiber content. As obtained in Section 4.3.1, the optimum asphalt binder content increased with the fiber content, which means more asphalt binder content would be required for the asphalt mixture mix design, thus the asphalt binder film thickness should be increased. Consequently, the interface adhesion between the fiber and asphalt binder would be improved and the moisture susceptibility performance would be improved also when the fiber content is smaller than 0.4 wt.%. However, once the content is over the optimal content, the excessive asphalt binder content may impair the mixture skeleton structure and reduce the mechanical strength under moisture conditions.

2. Freeze–thaw splitting test

**Figure 19:**
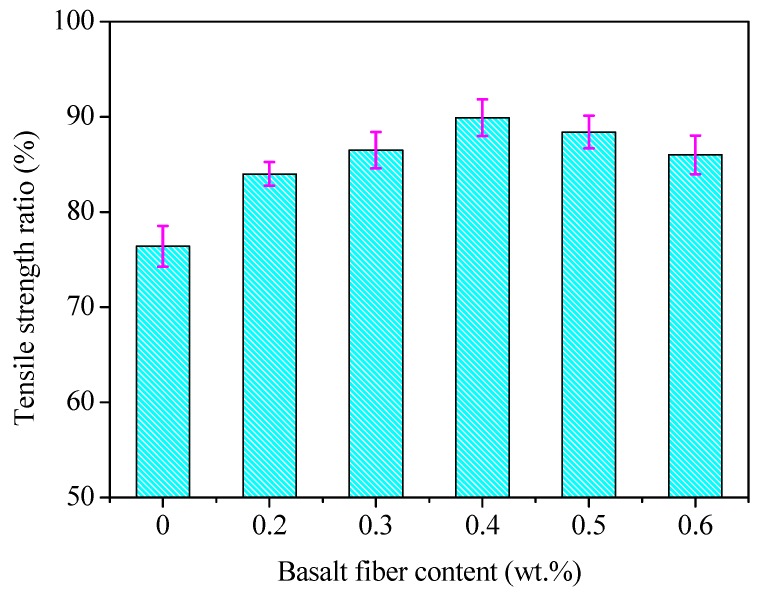
Tensile strength ratio of basalt reinforced asphalt mixture with different fiber contents.

The freeze–thaw splitting test was conducted for different fiber contents, and the results of the tensile strength ratio (TSR) are shown in Figure 19, where the error bar represents the standard variations of testing results.

Similar to immersion Marshall test results, the trend presented in Figure 19 shows that the *TSR* values increased initially and then decreased with the increase of basalt fiber content. The peak value of *TSR* was 89.9%, where the basalt fiber content was 0.4 wt.%. The reason for this is the same as the discussion in Section 4.3.3.

Combined with the results from the immersion Marshall test and freezing–thaw splitting test, the optimal fiber content could be obtained as 0.4 wt.%.

Overall, according to the results of the engineering properties from Section 4.3.2, Section 4.3.3, and Section 4.3.4, the standard deviations of the testing results (based on error bars) are within 20%, which meets the requirements of each specification. Combined with all the engineering properties, the optimal fiber content for basalt fiber-reinforced asphalt mixture is 0.4 wt.%.

## 5. Conclusions

The fiber type, fiber length, and fiber content as three key parameters, which significantly influence the fiber-reinforced asphalt mixture performance, were focused on in this study, and experiments on fibers, fiber-reinforced asphalt binder, and fiber-reinforced asphalt mixture were conducted. The main conclusions were obtained as following:

- Tests based on fiber properties of three kinds of fibers, namely basalt fiber, polyester fiber, and lignin fiber, showed that the favorable fiber type was basalt fiber. The same result was obtained based on comparisons among three kinds of fiber-reinforced asphalt binder performances.
- The results of the mesh-basket draindown test and cone penetration test for basalt fiber with three different lengths (6 mm, 9 mm, and 15 mm) showed that the favorable basalt fiber length was 6mm, when the mass loss in the mesh-basket draindown test was the lowest and the shear stress in the cone penetration test was the largest, relatively.
- The addition of basalt fiber can clearly improve the engineering properties of asphalt mixture, including high temperature stability, low temperature crack resistance, and water susceptibility, where the most favorable engineering performance will be presented under the fiber content of 0.4 wt.%, namely the optimum fiber content is 0.4 wt.%.

The following recommendations are suggested and can be conducted in the future:

- Basalt fiber with various lengths can be prepared to investigate the influence of fiber length on the performance of fiber-reinforced asphalt mixture and an optimal value can then be obtained.
- The reason why the optimum asphalt binder content increases with the content of fiber needs to be further verified.
- Field studies can be conducted to verify the conclusions obtained in this study.

## Figures and Tables

**Table 1:** Physical properties of asphalt binder.

Properties		Measured Values	Standard Value	Specification
Penetration (25 °C, 100 g, 5 s, 0.1 mm)		91	80–100	ASTM D5-97
Penetration index		−0.03	−1.5–1	N/A
Ductility (5 cm/min, 10 °C, cm)		40.4	≥30	ASTM D113-99
Softening point (°C)		48.5	≥45	ASTM D36-06
Viscosity (135 °C, Pa·s)		2.5	2.0–3.0	ASTM D341
Density (15°C, g/cm^3^)		1.017	–	ASTM D70-09
flash point (°C)		278	≥245	ASTM D92-02
Solubility (%)		99.8	≥99.5	ASTM D2042-81
After RTFOT *	Mass loss (%)	−0.30	≤ ±0.8	ASTM D2872-04
	Residual penetration ratio (%)	69.1	≥54	ASTM D5-97
	Ductility (5 °C, cm)	16	≥8	ASTM D113-99

* Rolling thin film oven test (RTFOT) conducted according to ASTM D2872-04.

**Table 2:** Properties of basalt fiber (provided by manufacturers).

Properties	Values	Specification
Density (g/cm^3^)	2.545	ASTM D3800
Heat resistance	180 °C, unchanged	N/A
Melting point (°C)	1500	ASTM D276
Corrosion resistance	High	N/A
Electrical conductivity	Low	N/A
Tensile strength (MPa)	≥3200	ASTM D2256
Modulus of elasticity (GPa)	≥96	ASTM C469
Fracture elongation (%)	3.2	ASTM D2256
Diameter (μm)	17	ASTM D2130

**Table 3:** Properties of polyester fiber (provided by manufacturers).

Properties	Values	Specification
Density (g/cm^3^)	1.364	ASTM D3800
Tensile strength (MPa)	≥1470	ASTM D2256
Fracture elongation (%)	6.0–8.0	ASTM D2256
Young modulus (GPa)	≥38	ASTM D638
Hot water resistance (°C)	≥104	N/A
Length (mm)	M ± 0.5	ASTM D204
Dispersivity (grade)	1–3	N/A
Diameter (μm)	24	ASTM D2130

**Table 4:** Properties of lignin fiber (provided by manufacturers).

Properties	Values	Specification
Density (g/cm^3^)	1.238	ASTM D3800
Fiber content	75%–80%	N/A
PH value	7.5 ± 1	N/A
Bulk density (g/L)	28–29	ASTM C29
Maximum fiber length (mm)	4.6 × 10^−2^	ASTM D204
Specific surface area (m^2^/g)	2.6	N/A
Diameter (μm)	49	ASTM D2130

**Table 5:** Physical and mechanical properties for three kinds of fibers.

Fiber Types	Lengths (mm)	Fracture Strength (MPa)	Color
Basalt fiber	6.1	3040	Brown
Polyester fiber	6.1	580	Milkiness
Lignin fiber	1.7	<320	Gray

**Table 6:** Thermostability for three kinds of fibers.

Fiber Types	Mass before oven Heated (g)	Mass after oven Heated (g)	Mass Loss (%)	Color Change
Basalt fiber	11	10.78	2	No
Polyester fiber	11	10.24	6.9	No
Lignin fiber	11	7.24	34	From gray to yellow

**Table 7:** Water absorption for three kinds of fibers.

Fiber Types	Mass before Water Absorption (g)	Mass after Water Absorption (g)	Water Adsorption (%)	Appearance Change
Basalt fiber	11	11.09	0.8	No
Polyester fiber	11	11.41	3.7	No
Lignin fiber	11	13.68	24.4	Apparent expansion

**Table 8:** Asphalt binder adsorption for three kinds of fibers.

Fiber Types	*m*_1_ (g)	*m*_2_ (g)	*m*_3_ (g)	Asphalt Binder Adsorption (%)
Basalt fiber	5	362.2	370.3	62
Polyester fiber	5	365.8	372.5	34
Lignin fiber	5	370.1	378.6	70
